# RE: Praluent (Alirocumab)-Induced Renal Injury

**DOI:** 10.1177/0897190017734429

**Published:** 2017-10-03

**Authors:** Robert S. Rosenson, Dominique Larrey, David D. Waters, Anders G. Olsson, Members of the Phase 3 Data Monitoring Committee for PRALUENT

**Affiliations:** 1 Icahn School of Medicine at Mount Sinai, New York, NY, USA; 2 Hepato-Gastroentérologie, CHU de Montpellier, Hôpital Saint-Eloi, Montpellier, France; 3 Division of Cardiology, San Francisco General Hospital, San Francisco, CA, USA; 4 Faculty of Health Sciences, Linköping University and Heart Center, Stockholm, Sweden

**Keywords:** alirocumab, rosuvastatin, acute renal injury

Jhaveri et al published a case report entitled “Praluent (Alirocumab)-Induced Renal Injury” in a recent issue of the *Journal of Pharmacy Practice*.^1^ The authors report a case of a 62-year-old female patient with chronic hypertensive nephropathy who experienced acute renal injury while treated with Praluent (alirocumab). While the information provided might be compatible with a possible causal role of alirocumab, several questions or comments can be raised/remain open for the discussion with the authors.

Although the patient had chronic kidney disease stage IV, she was treated with rosuvastatin 40 mg daily, which exceeds product indication by 4-fold.^2^ However, the possible role of rosuvastatin in inducing the acute renal injury has not been discussed. With regard to the hypothetical impact of alirocumab on serum creatinine levels, it appears from Figure 2 of the article that the increase in serum creatinine levels had started prior to the initiation of alirocumab. It would have been important to provide the evolution of this parameter over time rather than providing only the baseline and the maximum value. Also, other parameters such as creatine kinase values and urinary myoglobin, if available, would have been helpful to allow for additional assessment of the potential contribution to rosuvastatin-induced rhabdomyolysis and therefore kidney damage. In addition to rhabdomyolysis-induced acute kidney injury, rosuvastatin has been associated nonrhabdomyolysis causes of acute kidney injury. These nonrhabdomyolysis causes of acute kidney injury with rosuvastatin include renal tubular toxicity *and interstitial nephritis*.^3,4,5^ The frequency of acute kidney injury with statin therapy was reported in a large retrospective observational analysis of 7 administrative databases from Canada and 2 databases from the United Kingdom and United States which evaluated the risk of acute kidney injury in patients newly treated with high-potency statins that included rosuvastatin dosages ≥10 mg.^6^ Of more than 2 million new stain users, high-potency statin use was associated with a 1.34-fold (95% confidence interval: 1.25-1.43) higher fixed effect rate ratio of hospitalization for acute kidney injury than patients treated with low-intensity statin therapy.

It should be noted that the patient was also taking ramipril, a drug known to possibly play a role in the onset of acute renal injury. The information on the timing of action taken on ramipril in relationship to the onset of acute renal injury is missing. Regarding the medical history of this patient, the cardiovascular status of the patient was not characterized more precisely than by stating that the patient had a medical history of cardiac disease. No clear information was provided on the duration of the disease and on its renovascular expression. It remains unknown whether bilateral renal artery stenosis was excluded.

The authors propose a list of relevant alternative nondrug causes that they have ruled out; however, this list does not appear to be exhaustive. Other plausible causes of acute renal injury, such as infectious disease or ischemic causes, have not been evaluated. As already mentioned, the patient suffered from cardiac disease, without further diagnostic precision and without further information on her hemodynamic situation at the time of acute deterioration of renal dysfunction. It was unknown whether the patient had diabetes.

Another point to consider is that the authors mentioned, without providing precise values reported on a time scale, that the patient’s serum creatinine values returned to baseline about 6 weeks after the withdrawal of alirocumab. When considering the pharmacokinetic profile of alirocumab, it is relevant to note that 6 weeks after withdrawal of alirocumab, a residual level of the drug is known to be present in the patient’s body.

Finally, while assessing causality, the authors referred to the Naranjo algorithm yielding a score of 6 for a causal involvement of alirocumab in this case. Using the strict application of the information available in the article results in the score of 3, and this would be interpreted as “possibly related” rather than “probably related” to alirocumab. In addition, beyond this difference of scoring, it should be highlighted that the Naranjo algorithm has been previously challenged as a tool lacking validity and reproducibility in the attribution of causality, for example, in the assessment of hepatotoxicity.^7,8^ A more robust algorithm for causality assessment should therefore be considered, once the relevant missing information allowing a full assessment of this case has been made available by the authors.

We consider this case of renal injury resulted from excessive dosing of rosuvastatin, which has been previously associated with acute kidney injury. The temporal rise in serum creatinine occurred before initiation of alirocumab, supporting renal toxicity before the introduction of the PCSK9 inhibitor.
